# From Silence to Sound: Graeme Clark's Cochlear Implant

**DOI:** 10.7759/cureus.68580

**Published:** 2024-09-03

**Authors:** Riley Montgomery, Gauri Mankekar

**Affiliations:** 1 Otolaryngology, Louisiana State University (LSU) Health Shreveport School of Medicine, Shreveport, USA; 2 Otolaryngology, Otology - Neurotology, Louisiana State University (LSU) Health Shreveport School of Medicine, Shreveport, USA

**Keywords:** medical device innovator, cochlear impant, sensorineural (sn) hearing loss, bionic ear, historical vignette

## Abstract

Graeme Clark is an exceptional Australian professor and otolaryngologist who spent most of his life researching a way to help his deaf father and others suffering from profound hearing loss gain a better connection to those around them. His invention, the cochlear implant, has changed the lives of over 300,000 people around the world, with more than half of those individuals being children. Clark successfully created the first sensory connection linking the external world with human consciousness. He subsequently established the Bionic Ear Institute, now known as the Bionics Institute, to further improve his invention through research. He served as the full-time director until he retired at age 70 in 2005. Graeme Clark has been recognized worldwide for restoring hearing to the deaf and greatly improving their everyday lives.

## Introduction and background

**Figure 1:**
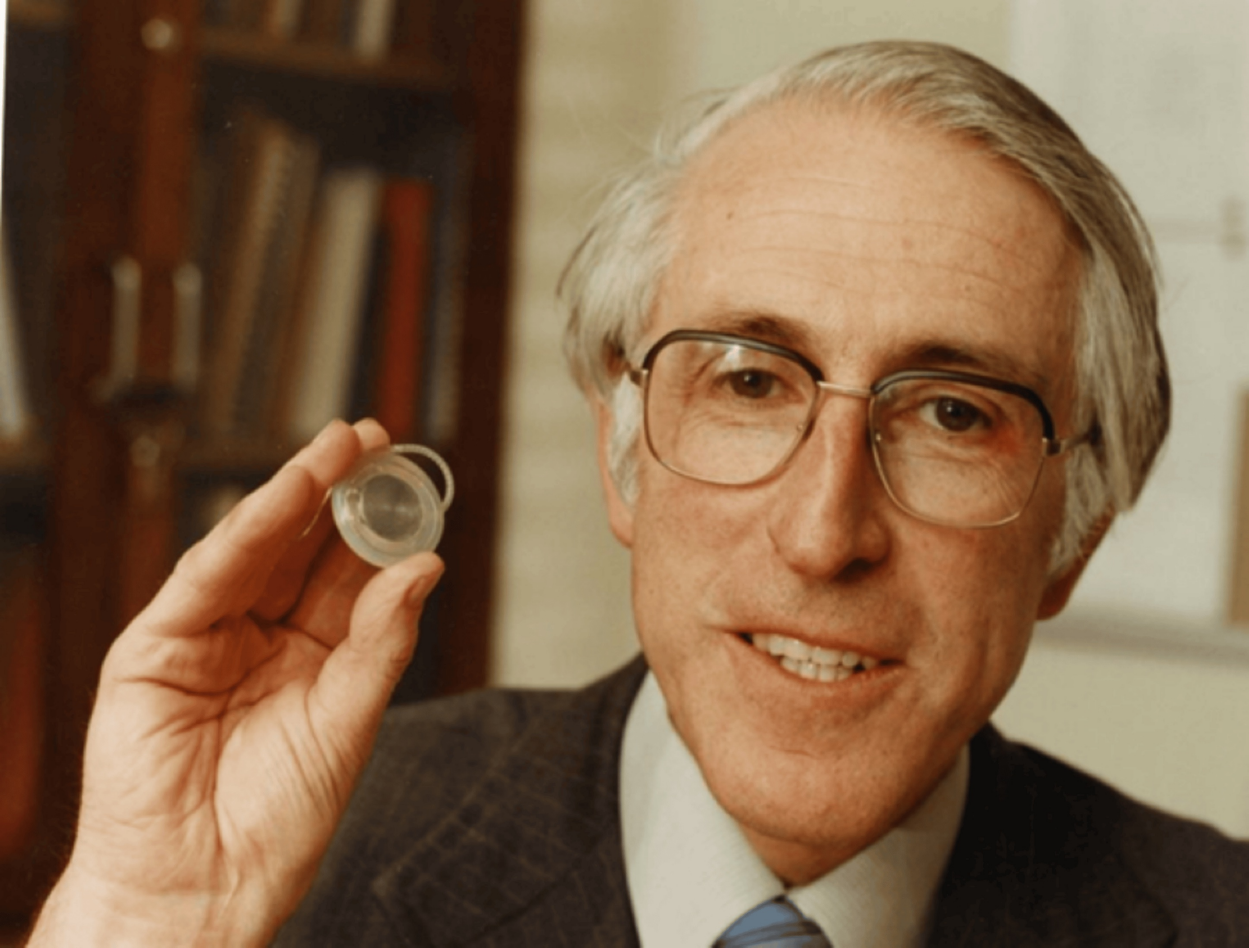
Graeme Clark with Cochlear's first multi-channel Nucleus implant, which he inserted in a patient in 1982. Source: Encyclopedia of Australian Science and Innovation. Licensed under the Creative Commons Attribution-Share Alike 4.0 International license. Courtesy of Graeme Clark [5].

Graeme Clark (Figure 1) is a world-renowned otolaryngologist and researcher. He and his team at the University of Melbourne, Australia, researched speech coding strategies and designed the multi-channel cochlear implant. Graeme Clark was born on August 16, 1935, in Camden, New South Wales, Australia. His father was a pharmacist with sensorineural hearing loss, which initially inspired Clark to study the ear. Clark observed the difficulty his father had communicating with those who came into his pharmacy, often having to ask people to speak up [1]. At a young age, he understood the challenges of daily living for the deaf community and became determined to make a change [2,3]. At just 10 years old, Clark told his local minister that he wanted to be an ear doctor when he grew up. In 1957, he earned his medical degree from the University of Sydney, followed by a fellowship at the Royal College of Surgeons, London. He returned to Australia and held the position of senior ENT surgeon from 1963 to 1966 [1,4]. Soon after, Clark began researching the possibility of an electronic, implantable hearing device in the hopes of restoring hearing to deaf individuals. He returned to study and completed his PhD thesis at the University of Sydney on "Middle Ear & Neural Mechanisms in Hearing and in the Management of Deafness." From 1970 to 2004, he held the William Gibson Chair of Otolaryngology at the University of Melbourne [1]. In 1999, he was appointed laureate professor at the University of Melbourne.

## Review

Cochlear implant

By the mid-1960s, Graeme Clark’s desire to help deaf individuals became a reality. He read a scientific paper by Dr. F. Blair Simmons, Professor of Surgery and Otolaryngology, describing how the deaf could hear via electrical stimulation. However, understanding speech remained a barrier. Clark soon gave up his senior surgical position to commit his life to research in auditory brain science, including speech recognition. In 1967, Graeme Clark began exploring the potential for an electronic, implantable hearing device, while acknowledging the importance of safety [2,3]. Many people were concerned about the risk of meningitis, tissue damage, and other hazards associated with placing a current in the human body [6,7]. Clark was challenged by many of his colleagues, who argued that his idea was futile, his research was fabricated, and that operating on the inner ear would be reckless. Of course, Clark proved them wrong [3,7]. One fundamental moment in his research came when he was at the beach, playing with a shell that resembled the structure of the cochlea. He realized that a piece of grass, if flexible at the tip, could traverse the cochlea’s turns, just as an electrode would need to do. The first bionic ear prototype was implanted in 1978 after Clark and his team discovered how speech could be coded using multiple electrical signals [2]. The following year, he received a grant to fund commercial development, and by 1982, he had completed the first successful clinical trial [1]. Cochlear implants were approved by the FDA for adult patients in 1985 and for children 17 and under in 1990. Research on the cochlear implant has continued in the hopes of improving the device. At the end of 2012, the National Institutes of Health (NIH) estimated there to be about 324,200 individuals with cochlear implants [6,8].

Impact and legacy

When hearing fades for individuals, so does the world around them. Although Clark’s father had a major impact on his aspirations in life, he was primarily motivated by deaf children since they had their entire lives ahead of them [1]. Childhood deafness leads to the inability to understand language and, therefore, to speak, limiting educational opportunities and ultimately career choices. Less than a generation ago, deaf individuals had no hope of ever hearing again. Thanks to Graeme Clark and the cochlear implant, deaf individuals, particularly children, now have a much better outlook on life. The World Health Organization estimates that about 360 million people worldwide suffer from profound hearing loss [7]. It is arguably one of the most important devices for assisting deaf and hearing-impaired children in communicating since the invention of sign language. As of 2022, one million cochlear implants have been placed worldwide [9]. Clark established the Bionic Ear Institute in 1986 and served as the full-time director until 2005. In 2009, the Bionic Ear Institute became the Bionics Institute, which now not only focuses on advancing the cochlear implant but also creates medical devices for other neurosensory disorders like blindness and paraplegia [4,5,10,11].

In his 2012 article, Clark recounted the work done by him and his team at the University of Melbourne that led to the development of the multi-channel cochlear implant and its subsequent commercial development by Cochlear Limited [12]. In 1978, they discovered the first speech coding strategy to provide open-set speech understanding. It was the first interface between the world and human consciousness to be successful in a clinical environment. In 1989, he performed a bilateral implant [8,12]. The speech perception from the initial group of patients showed that the auditory stimuli from each side could be fused into a single image and localized depending on the intensity of the sound and its timing of arrival. He implanted his first pediatric patient in 1985 with a multi-channel cochlear implant. This led to the finding that the age of the child at surgery determined the development of speech understanding and spoken language, with improved results being seen when the child was younger than 12 months [12]. In 1990, the FDA approved the first cochlear implant in children. Soon, Clark and his team reported that bimodal speech processing using a cochlear implant in one ear and a hearing aid in the other ear was feasible [8]. Today, this strategy is used for hearing rehabilitation worldwide.

Awards and honors

Soon after founding the Bionic Ear Institute, Clark began receiving numerous awards and honors, which continue to this day. Initially, he was recognized locally and was awarded the James Cook Medal from the Royal Society of New South Wales in 1991, the Sir William Upjohn Medal from the University of Melbourne in 1997, and the Australian Prime Minister’s Prize for Science in 2004. Thereafter, Clark received various other prestigious medals, including the Zulch Prize from the Max Planck Institute in 2007 (Germany’s highest prize in neuroscience) and the Lister Medal in 2010 (considered the most prestigious award in the world for surgical science). Aside from various awards, Clark is also a Fellow of the Australian Acoustical Society, a Fellow of the Australian Academy of Science, a Fellow of the Royal Society of London, an Honorary Fellow of the Royal Society of Medicine, and an Honorary Fellow of the Royal College of Surgeons, among many others [4,13]. In 2013, Professor Clark was awarded the prestigious Lasker-DeBakey Clinical Medical Research Award along with two other cochlear implant pioneers, Ingeborg Hochmair (MED-EL) and Professor Blake Wilson, Duke University [14].

## Conclusions

Graeme Clark’s desire to help the deaf and his dedication to finding a way to restore their hearing has changed the lives of thousands of people around the world. The cochlear implant is recognized as a landmark discovery in medical science, being the first significant improvement for the deaf community since the invention of sign language over 250 years ago. Not only has his invention restored hearing to those suffering in silence, but it has also given them opportunities to form relationships, gain meaningful employment, and ultimately be successful in the world. His perseverance and dedication did not end with the first successful implant, as he continued researching to improve it. He has also been an inspiration to many, paving the way for other researchers to continue seeking ways to bring hope and restore life to those with other neurosensory disorders.

## References

[1] Professor Graeme Clark, Otolaryngologist (Interview by S. O'Leary) [Transcript]. (2011 (2024). Professor Graeme Clark, otolaryngologist (interview by S. O'Leary). https://www.science.org.au/learning/general-audience/history/interviews-australian-scientists/professor-graeme-clark.

[2] Ho JP, North H, Singh NP (2018). Forty years of "Waltzing Matilda": the history of the multichannel cochlear implant. Med J Aust.

[3] Bondarew V, Seligman P (2012). The Cochlear Story. The Cochlear Story.

[4] (2024). Pioneer of the bionic ear. Bionics Institute. https://www.bionicsinstitute.org/laureate-professor-graeme-clark-ac/.

[5] (2024). Encyclopedia of Australian Science and Innovation person Clarke Graeme M. https://www.eoas.info/biogs/P001420b.htm.

[6] Clark G (2003). Cochlear implants in children: safety as well as speech and language. Int J Pediatr Otorhinolaryngol.

[7] O'Donoghue G (2013). Cochlear implants - science, serendipity, and success. N Engl J Med.

[8] Clark GM (2015). The multi-channel cochlear implant: multi-disciplinary development of electrical stimulation of the cochlea and the resulting clinical benefit. Hear Res.

[9] Zeng FG (2022). Celebrating the one millionth cochlear implant. JASA Express Lett.

[10] Clark G (2017). Creating the bionic ear: the central role of cybernetics [Leading edge]. IEEE Technol Soc Mag.

[11] (2024). Our history. https://www.bionicsinstitute.org/about/our-history/.

[12] Clark G (2012). The multi-channel cochlear implant and the relief of severe-to-profound deafness. Cochlear Implants Int.

[13] (2024). The University Sydney School of Medicine Online Museum - Clark Graeme M. https://www.sydney.edu.au/medicine/museum/mwmuseum/index.php/Clark.

[14] (n.d.). Modern cochlear implant. https://laskerfoundation.org/winners/modern-cochlear-implant/.

